# Three‐Dimensional Printing of Cardiac Reconstruction, Facilitating Customized Closure Device of Gigantic Left Atrial Appendage: Case Report

**DOI:** 10.1155/cric/7409635

**Published:** 2026-09-07

**Authors:** Mann Chandavimol, Tawai Ngernsritrakul, Krissada Meemook, Kakanand Srungboonmee, Thinnakrit Sasiprapha, Thanakhom Hoontrakul

**Affiliations:** ^1^ Division of Cardiology, Department of Internal Medicine, Faculty of Medicine, Ramathibodi Hospital, Mahidol University, Bangkok, Thailand, mahidol.ac.th; ^2^ Center for Research Innovation and Biomedical Informatics, Faculty of Medical Technology, Mahidol University, Bangkok, Thailand, mahidol.ac.th; ^3^ Thammasat University Hospital, Thammasat University, Pathum Thani, Thailand, tu.ac.th

**Keywords:** cardiac reconstruction modeling, case report, customized left atrial appendage occlusion device, giant left atrial appendage, three-dimensional printing

## Abstract

We present a successful case of 3D printed cardiac reconstruction for a custom‐made LAAO LAmbre (Lifetech Scientific Corp., Shenzhen, China) device of gigantic and challenging LAA anatomy.

## 1. Introduction

In patient with atrial fibrillation (AF), left atrial appendage (LAA) is a common source of the thrombus. The standard treatment is oral anticoagulant (OAC) but it exposes patients to hemorrhage risk [1]. Multiple trials have showed left atrial appendage occlusion (LAAO) is safe and effective in preventing AF related thromboembolism in high bleeding risk population [2–4] as well as in patient with absolute contraindication for OAC [5].

A giant LAA with complex anatomy may impedes the implantation of a conventional LAAO device, resulting in device embolization or peridevice leakage. The leakage has been associated with thromboembolic event, necessitating a complete closure [6]. Consequently, routine incorporation of CT was associated with excellent outcomes for procedural safety and absence of major residual leak [7] and Hell et al. demonstrated that CT‐based 3D printing models may assist device selection and prediction of device compression [8]. Inohara et al. reported using custom‐made LAAO device on gigantic LAA in Canada. We wish to demonstrate the successful transcatheter custom‐made LAAO device with 3D printing cardiac reconstruction modeling in Asia.

## 2. Case History

A 69‐year‐old Thai male with medical history significant for diabetes mellitus, hypertension, dyslipidemia, chronic kidney disease Stage IIIa, sick sinus syndrome status post (S/P) dual‐chamber pacemaker implantation, stable coronary artery disease S/P percutaneous coronary intervention (PCI) of the proximal left anterior descending artery 6 months prior to admission, paroxysmal AF S/P radiofrequency ablation (RFA) twice, history of LAA thrombus and previous cerebrovascular accident. He also had a history of recurrent upper gastrointestinal bleeding admission due to *H. pylori* infection, required blood transfusion. H*. pylori* infection was successfully eradicated using a full course of antibiotics at another medical center. A hematologic investigation concluded patient suffered from idiopathic thrombocytopenic purpura with platelet count ranging 70,000–120,000/uL. There is no immunosuppressant initiated. His calculated stroke cardioembolic and bleeding risk were CHA_2_DS_2_‐VASc = 3, and HAS‐BLED = 4.

### 2.1. Antithrombotic History

Patient was originally on Warfarin when diagnosed with AF, then developed upper gastrointestinal bleeding. Dabigatran was then initiated. The dose of dabigatran was reduced due to impaired kidney function. Clopidogrel was started after PCI, which was performed about 6 months before the LAA closure. He was on dual antithrombotic treatment (dabigatran plus clopidogrel) for 6 months. At the time of LAA closure, patient was only on dabigatran 110 mg BID.

The multidisciplinary team proposed a transcatheter LAAO as an alternative to antithrombotic treatment for stroke prevention.

### 2.2. Investigation and Treatment

**Figure 1:**
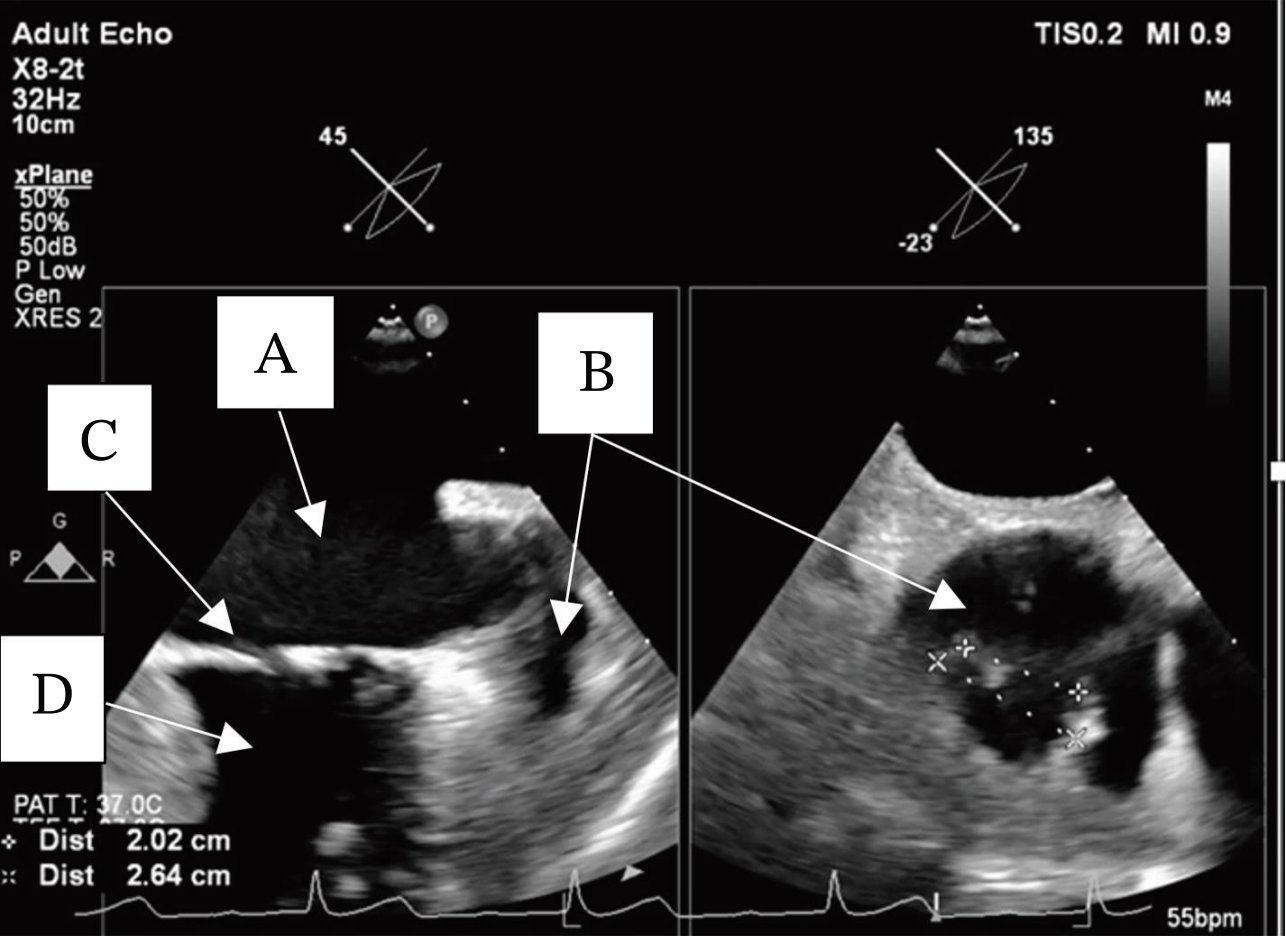
Echocardiographic images of left atrial appendage in 45° and 135° short axis view. The 45° short axis view shows (A) left atrium, (B) left atrial appendage, (C) mitral valve, and (D) left ventricle. The 135° short axis view shows (B) left atrial appendage.

**Figure 2:**
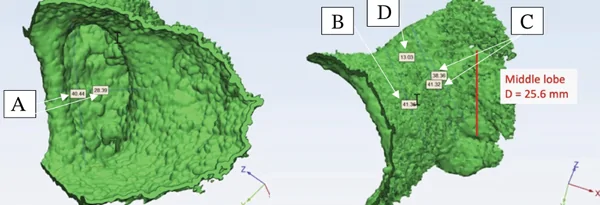
3D CT reconstruction of left atrial appendage in long axis and short axis views (A) Ostium 40.44 mm (long axis) and 28.39 mm (short axis), (B) ostium 41.36 mm with landing zone (C) 38.36–41.32 mm, and (D) length between ostium and landing zone of 13.03 mm (posterior to ostium).

The initial imaging of transesophageal echocardiogram (TEE) found a 3‐lobed cauliflower‐type LAA with very large oval‐shaped ostium of 26–42 mm in diameter, landing zone of 24–42 mm, and depth of 36 mm as shown on Figure 1. Three‐dimensional (3D) computered tomography (CT) (Phillips, Andover, Massachusetts) with 381 mA x‐ray tube and KVP 140 kV was used for detailed evaluation of the complex 3‐lobe LAA anatomy as seen on Figure 2.

**Figure 3:**
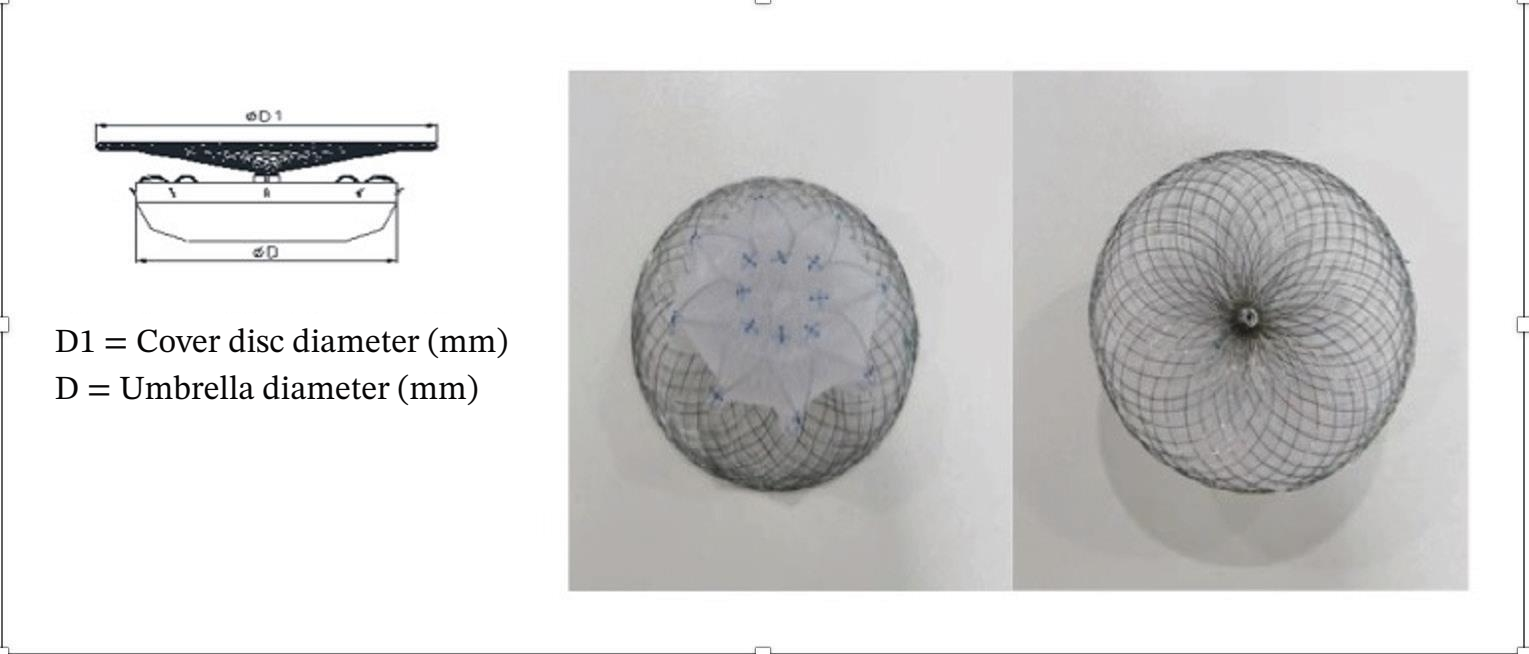
Design of the custom left atrial appendage occlusion device (LAmbre): D = 30 mm umbrella size and D1 = 44 mm covering disc size.

Given the patient′s extremely large LAA size, no commercially available device was sufficient to accommodate its size, as the largest available Watchman (Boston Scientific, Marlborough, United States), Amulet (Abbott, Illinois, United States), and LAmbre (Lifetech Scientific Corp., Shenzhen, China) devices were 35, 34, and 40 mm in diameter, respectively. The LAmbre device is a self‐expanding LAA occluder composed of a nitinol mesh and polyester membranes, featuring an umbrella and a cover connected by a short central waist. The advantage of the product is its ability to customize both the umbrella and a cover independently. After a careful review, the manufacturer was agreeable to make the prosthesis by using CT data in determining optimal prosthesis position and sizing by identifying new optimal landing zone and ostium. In this case, the middle lobe is the largest and likely to be the most stable landing area, measured 25.6 mm with the LAA opening of 41.3 mm in diameter, instead of the traditional superior lobe anchoring; a custom‐made 30/44 mm LAmbre LAAO device (30 mm umbrella size and 44 mm covering disc size) for middle‐lobe anchoring was created in accordance. Figure 3.

The 3D printed cardiac reconstruction was assembled for a clearer understanding of LAA anatomy and device testing. 3D printing was done by acquiring data from gated cardiac CT using the slice thickness of 0.67 mm with 0.335 mm slice increment for total of 423 slices with blood pool contrast of the left atrium. The best systole image is individuated and imported into Mimics 26.0 (Materialise, Leuven, Belgium) for image segmentation in DICOM file for total cardiac reconstruction model. Then 3‐Matic 18.0 (Materialise, Leuven, Belgium) was used for 3D LAA image isolation. The model was printed with a fused deposition modeling 3D printer (Flash Forge Dreamer, Zhejiang Flashforge 3D Technology Co. Ltd) using ABS filament. The printing process was 6 h 48 min with 30.5 g of material. Subsequently, the support structures of the printed model were carefully excised to reveal the final LAA anatomy.

**Figure 4:**
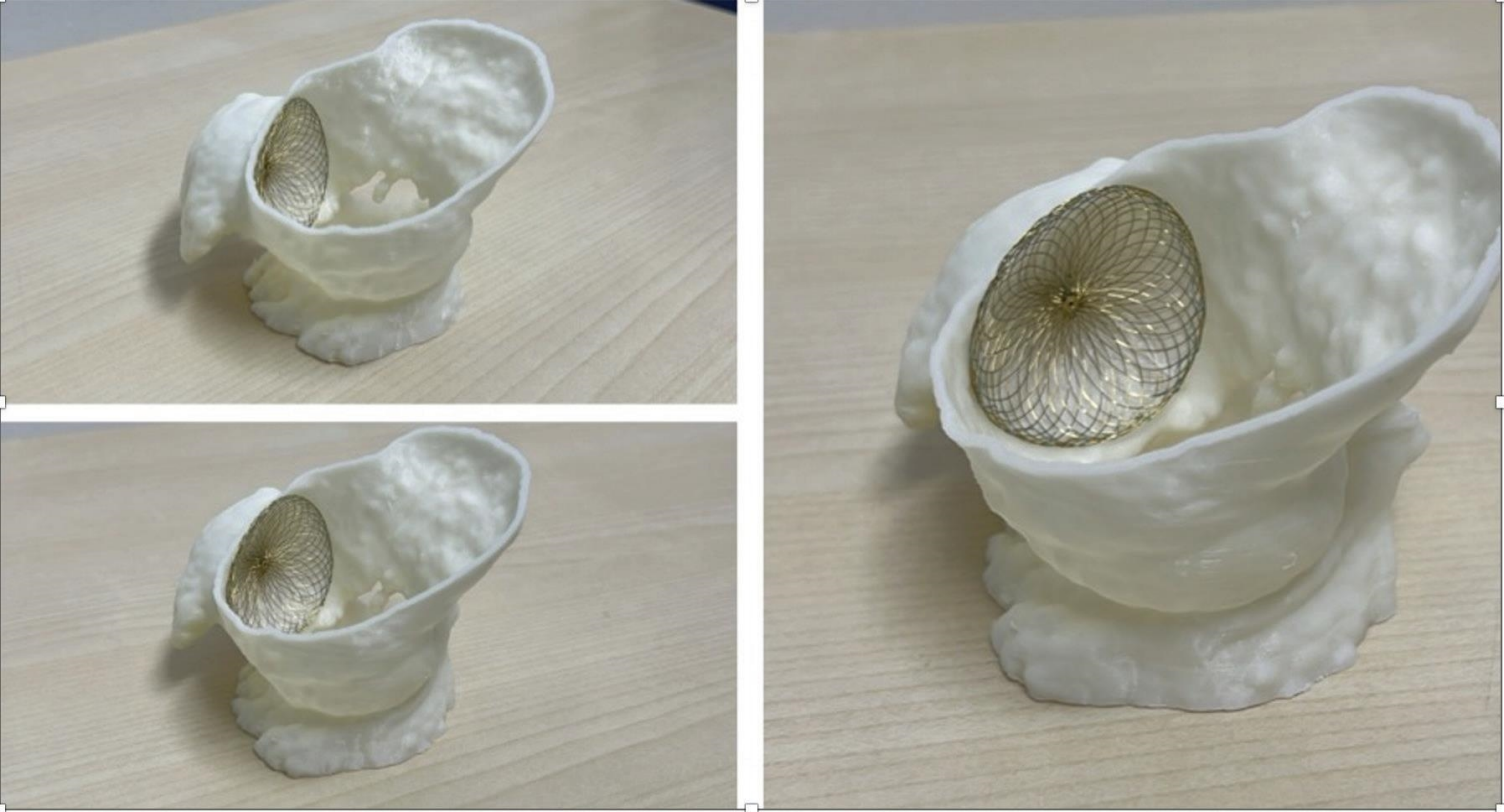
3D printing of cardiac reconstruction of left atrial appendage with custom LAmbre device implantation rehearsal.

Figure 4 shows testing on the cardiac reconstruction model for proper procedural planning of implantation technique and suitability of the anatomy.

After receiving approval from our local heart team, the case was scheduled for the procedure through compassionate use facilitated by our hospital and local regulatory authorities. The additional cost of the device, which was twice the cost of the regular device, was covered by the patient through self‐payment. From the time of CT acquisition to device size and deployment planning strategy, approximately 2 months were required. The custom device manufacturing process also took around 2 months, followed by an additional 2 months for compassionate entry approval. The total duration from CT acquisition to device implantation was approximately 6 months.

**Figure 5:**
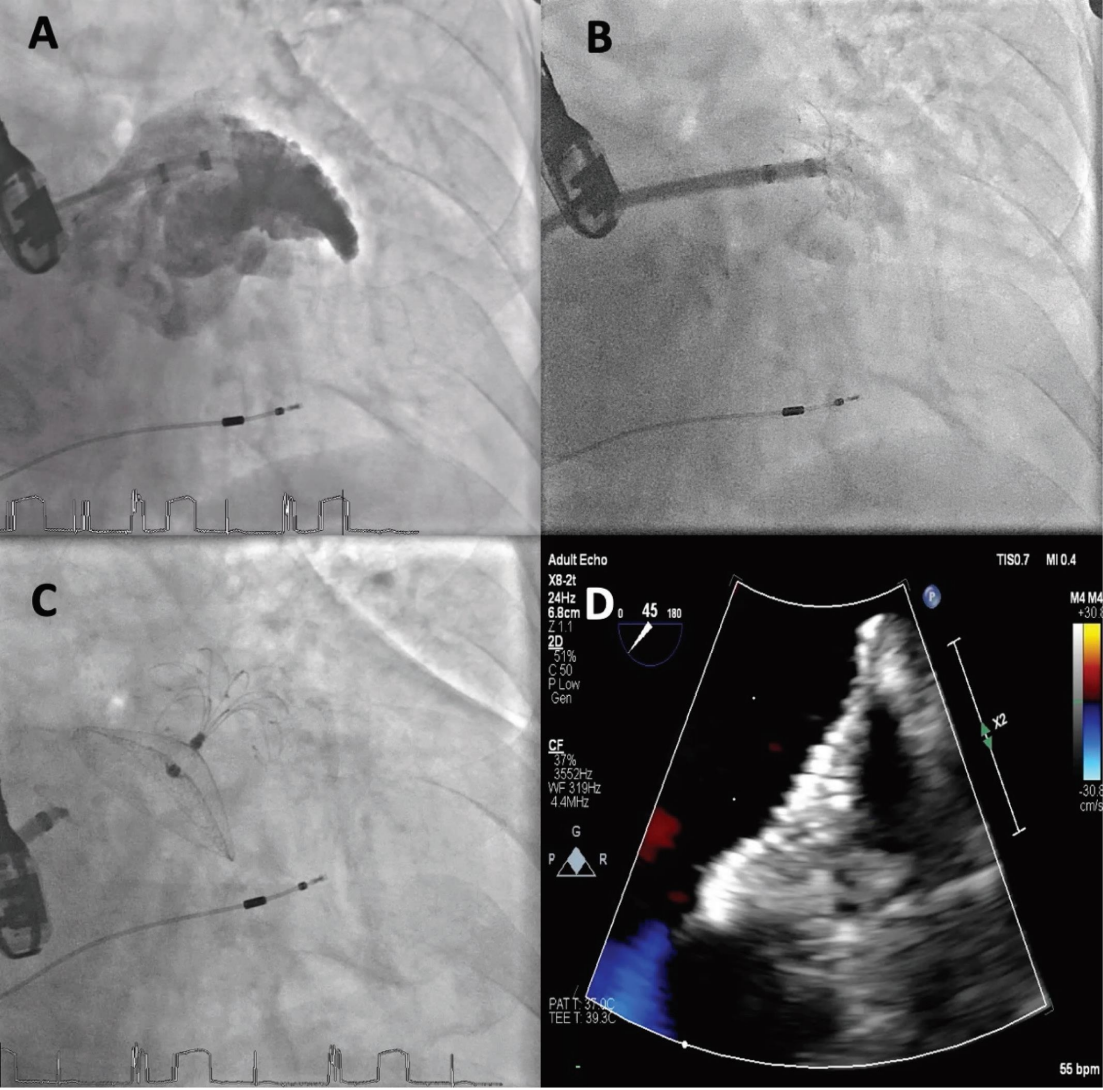
(A) LAA middle lobe angiography, (B) device deployment using umbrella for anchoring, (C) successful LAmbre device deployment, and (D) intraprocedural TEE confirmed LAmbre device in optimal position with no peridevice leakage.

Transcatheter LAAO procedure was then performed under general anesthesia with TEE and fluoroscopic (General Electric Healthcare Discovery IGS 730, Chicago, United States) guidance. Right femoral venous access and transeptal puncture through 10 French delivery system was done. The custom‐made LAmbre device 30/44 mm was successfully delivered. In fact, in light of the gigantic LAA complex anatomy, an anatomic specific implantation technique was employed to anchor the device at the middle‐lobe instead of the standard superior lobe anchor as mentioned earlier. Intraprocedural TEE revealed suitable positioning without peridevice leakage and complications Figure 5.

The patient was discharged 1‐day postimplantation with home‐medication of dabigatran 110 mg twice daily. Additionally, a 60‐day TEE follow‐up showed no peridevice leakage and no mitral valve injury. Dabigatran was then discontinued after 2 months and aspirin 81 mg/day for clinical indication of stable CAD post PCI.

The patient has been under follow‐up for 4 years to date, during which time his general cardiologist transitioned him to clopidogrel monotherapy. The patient has remained free of upper gastrointestinal bleeding, transfusions, thromboembolic events, and device‐related thrombus. A TEE conducted at the 4‐year revealed the device to be in a stable position, with the device disc showing endothelialization and no peridevice leakage. File S1, S2, S3, and S4.

## 3. Conclusion

In conclusion, we successfully implanted a custom‐made LAAO LAmbre (Lifetech Scientific Corp., Shenzhen, China) device with middle‐lobe anchoring in a challenging gigantic, three‐lobed cauliflower LAA. Our case also demonstrates the use of 3D printing for cardiac reconstruction to aid the design and test the custom‐made device to ensure optimal results.

## 4. Discussion

Clinically, our patient had persistent AF with a high CHA_2_DS_2_‐VASc, exposing the patient to a high thromboembolic risk. Unfortunately, the patient also suffered from episodes of bleeding and a high HAS‐BLED score, hindering long‐term OAC therapy. Our patient was recommended the LAAO procedure in pursuant to the current treatment guideline [9, 10].

Our case highlights the successful implantation of a custom‐made 30/44 mm LAAO LAmbre device with 3D printed cardiac reconstruction modeling of giant cauliflower LAA. In fact, Pellegrino et al. reported a case of using a 3D printed cardiac model to rehearse the procedure [11] and another case report from Barocelli et al. used a 3D CT to guide the manufacturing of a custom‐made LAAO device [12]. Kim et al. demonstrated that 3D printed models showed superior agreement to the implanted LAAO sizing than 2D TEE [13]. In addition, our complex gigantic LAA anatomy stresses the importance of careful procedural planning. We also used 3D printed cardiac model to test the custom‐made device with middle‐lobe anchoring for comprehensive understanding and implantation technique adaptation. However, rigid ABS 3D‐printed model may not accurately reproduce LAA compliance, wall deformation, or tissue‐device interaction. Consequently, although the model may help assess anatomical feasibility and device orientation, it cannot reliably predict in vivo device stability or migration risk.

The regional prevalence of LAA morphologies has significant clinical implications, particularly in the Asian population, where the cauliflower‐type LAA is more common compared with the windsock type prevalent in Western populations [14]. This distinction is critical, as research by Simon et al. has shown that the windsock morphology serves as a protective factor against stroke relative to the cauliflower type [15]. Additionally, LAAO remains a topic of debate regarding its hemodynamic effect. Although a pilot study by Ortiz‐Leon et al. [16] reported no significant impact on left upper pulmonary vein pressure post‐LAAO, a meta‐analysis by Gofus et al. [17] highlighted conflicting evidence regarding the procedure′s hemodynamic effects. This underscores the need for tailored approaches in managing patients with varying LAA morphologies, particularly in populations with a higher prevalence of the cauliflower type, to optimize stroke prevention and maintain hemodynamic stability.

Finally, a short‐term OAC therapy was prescribed to our patient to prevent device‐related thrombosis, which shown to be safe as previously reported [18].

## Author Contributions

Conceptualization and validation: Mann Chandavimol, MD and Kakanand Srungboonmee, PhD. Data curation and formal analysis: Mann Chandavimol, MD; Tawai Ngernsritrakul, MD; Krissada Meemook, MD; Kakanand Srungboonmee, PhD; and Thinnakrit Sasiprapha, MD. Investigation and methodology: Mann Chandavimol, MD; Tawai Ngernsritrakul, MD; Krissada Meemook, MD; Kakanand Srungboonmee, PhD; Thinnakrit Sasiprapha, MD; and Thanakhom Hoontrakul. Project administration: Mann Chandavimol, MD and Thanakhom Hoontrakul. Resources, software, and visualization: Mann Chandavimol, MD; Kakanand Srungboonmee, PhD; and Thanakhom Hoontrakul. Supervision: Mann Chandavimol, MD. Writing—original draft: Thanakhom Hoontrakul. Writing—review and editing: Mann Chandavimol, MD and Thanakhom Hoontrakul.

## Funding

No funding was received for this manuscript.

## Disclosure

Mann Chandavimol was a proctor for LAmbre device.

## Ethics Statement

The Internal Review Board had approved this case report (COA. No. MURA2024/472). The authors are accountable for all aspects of the work in ensuring that questions related to the accuracy or integrity of any part of the work are appropriately investigated and resolved. All procedures performed in this study were in accordance with the ethical standards of the institutional and/or national research committee(s) and with the Helsinki Declaration (as revised in 2013).

## Consent

Written informed consent was obtained from the patient for the publication of this case report and accompanying images. A copy of the written consent is available for review by the editorial office of this journal.

## Conflicts of Interest

The authors declare no conflicts of interest.

## Supporting Information

Additional supporting information can be found online in the Supporting Information section. Files S1–4: Follow‐up transesophageal echocardiography result at 4 year.

## Supporting information


**Supporting Information 1** File S1: LAmbre device in optimal position in 3‐dimension imaging.

**Supporting Information 2** File S2: LAmbre device at 0 and 90°.

**Supporting Information 3** File S3: LAmbre device at 45 and 135°.

**Supporting Information 4** File S4: LAmbre device showed no paradevice leakage.

## Data Availability

The data that support the findings of this study are available on request from the corresponding author. The data are not publicly available due to privacy or ethical restrictions.
